# Investigation of the effect of thymoquinone and doxorubicin on the EGFR/FOXP3 signaling pathway in OVCAR3 human ovarian adenocarcinoma cells

**DOI:** 10.1590/acb401725

**Published:** 2025-03-31

**Authors:** İlhan Özdemir, Ayfer Şanli Aktaş, Mehmet Cudi Tuncer

**Affiliations:** 1 Atatürk University – Faculty of Medicine – Department of Gynecology and Obstetrics – Erzurum – Turkey.; 2 Dicle University – Faculty of Medicine – Department of Histology and Embryology – Diyarbakir – Turkey.; 3 Dicle University – Faculty of Medicine – Department of Anatomy – Diyarbakir – Turkey.

**Keywords:** Ovary, Antioxidants, Epidermal Growth Factor Receptor, Forkhead Box Protein O3, Apoptosis, Cell Movement

## Abstract

**Purpose:**

To investigate the cytotoxic and apoptotic effects of the combination of doxorubicin (Dox) and thymoquinone (TQ) on ovarian adenocarcinoma cells (OVCAR3) via the EGFR/FOXP3 signaling pathway.

**Methods:**

We used human OVCAR3 and human skin keratinocyte cells (HaCaT). Different concentrations of TQ and Dox were applied to the cells for 24, 48, and 72 hours, and the cytotoxicity level was determined via the MTT method. Expression levels of EGFR/FOXP3 for cell proliferation and apoptosis were determined by quantitative reverse transcription polymerase chain reaction (RT-qPCR) and Western blot analysis. The colony counting was performed after DAPI staining, and the effect on cell proliferation was determined.

**Results:**

Cytotoxicity was found to be highest with TQ and Dox treatments, and cell migration was prevented, especially in the group that received combined TQ and Dox treatment. Moreover, using RT-qPCR analysis, activity in the EGFR and FOXP3 pathway was found to be downregulated the most with TQ, and the amount of protein decreased with TQ and Dox.

**Conclusions:**

The findings showed that the greatest cytotoxic effect and the most apoptosis occurred during TQ treatment. Additionally, it was determined that a significant decrease in EGFR and FOXP3 levels occurred with the application of TQ and Dox.

## Introduction

Ovarian cancer causes the most deaths among gynecological diseases, and it is known that tens of thousands of women die from this cancer every year; it ranks fifth in cancer-related deaths in women^1^. Most epithelial ovarian cancer cases show that peritoneal carcinomatosis and abdominal ascites are common findings and characteristics when metastatic disease is diagnosed^2^. Innovative treatment methods have been developed for the early diagnosis and treatment of this disease; thus, the mortality rate from this cancer can be significantly reduced^3^. Surgery and chemotherapy are the most common methods used in ovarian cancer treatment^4^. When treatment methods for ovarian cancer are determined and applied, it appears to be resistant to chemotherapy when it reoccurs^5^.

Generally, cisplatin application is the most common treatment modality for patients who are resistant in advanced stages^6^. In many different types of cancer, excessive activity caused by oncogenic factors also eliminates the activity of suppressor genes that control tumor proliferation. Therefore, when we consider this as a new approach in cancer, increasing the expression of these genes by turning to genetic and epigenetic factors plays a positive role in the apoptosis process, which is of great importance for ovarian and other cancers^7^.

Considering the studies carried out in recent years, the increase in studies and information on medicinal plants effective in cancer resulted in an increase in the treatment methods for cancer types^8^. Studies show that thymoquinone (TQ), which is observed as an effective weapon, especially in cancer, is a product of *Nigella sativa*, which is a ray of hope in preclinical treatments for cancer patients and is known to have antitumor activity. Moreover, TQ has numerous molecular mechanisms of action that have been proven to prevent tumor growth and prolong the life of cancer patients. In addition, TQ, known as the major bioactive compound in black cumin essential oils, is of great importance in cell culture and in-vivo disease models, and it has been reported to exhibit promising pharmacological and therapeutic effects in treatment studies^9^.

The epidermal growth factor receptor (EGFR) is a transmembrane glycoprotein that can regulate cell proliferation and signaling pathways^10^. The EGFR gene is located on chromosome 7p12-13 and belongs to the cell membrane receptor tyrosine kinase family, including EGFR (erbB1), erbB2 (HER2), erbB3 (HER3), and erbB4 (HER4). Among these members of the HER family, EGFR and HER2 are the most frequently altered receptors in cancer^11^. Although EGFR is expressed in 90% of certain subtypes of ovarian cancers, this molecule has a response rate of 0–6% in patients with persistent or recurrent disease, preventing it from being a therapeutic target and a potential prognostic biomarker in ovarian cancer^12^, as it lacks meaningful survival qualities. It provides benefits to patients who respond positively to chemotherapy or whose disease is stable^13^.

In this study, a cell line (ovarian adenocarcinoma cells–OVCAR3) belonging to the epithelial subtype, one of the most common types of ovarian cancer, was used. The anti-cancer effects of single and combination application of doxorubicin (Dox) and TQ compound were evaluated. Decreasing long-term survival rate and recurrence have increased the interest in alternative drugs that are less toxic and more anticancer effective for ovarian cancer.

## Methods

### Materials

NIH:OVCAR-3 (HTB-161^TM^) and HaCat (RRID:CVCL_0038) were used as biological materials. The chemicals used were TQ (274666-1G, Sigma), Dox (Koçak Pharma, Türkiye), phosphate buffer salt (PBS), 0.05% trypsin–ethylene diamine tetraacetic acid (Trypsin-EDTA), Roswell park memorial institute medium (RPMI), glutamine and penicillin-streptomycin solution, fetal bovine serum (FBS) (Gibco, United States of America), dimethyl sulfoxide (DMSO) (Merck, United States of America), and Dulbecco’s modified eagle medium (DMEM) (Invitrogen, USA). The TQ used in this study was provided with 98% purity.

### Cell culture

The OVCAR-3 cell line was cultured in RPMI 1640 medium, and the HaCaT cell line was cultured in DMEM. The medium used in our study was RPMI 1640 medium, which contains 10% FBS, 1% L-glutamine, penicillin (100 U/mL), and streptomycin (100 µg/mL). The viability, proliferation, passage, and follow-up processes of the cells were monitored using an inverted microscope. The cells were incubated under conditions of 95% humidity and 5% CO2 at 37°C.

### MTT assay (cell viability) and IC_50_

TQ and Dox were applied to ovarian cancer cells at different concentrations, and the MTT cell proliferation test was performed to determine cell viability and the concentration at which 50% of the cells survived (IC_50_). MTT Yellow tetrazolium MTT (3-(4, 5-dimethylthiazolyl-2)-2,5-diphenyltetrazolium bromide) is a water-soluble substance and turns orange when reduced to Formazione components in living cells. Various concentrations of TQ were dissolved in DMSO at a ratio of 1/1,000 in a complete medium containing 10% serum, and a 50 mM stock solution was prepared and used.

Cells were seeded in a plate at the concentration of 5×10^3^ OVCAR-3 cells in each well and in 100 µL of RPMI growth medium. The cells were kept in an incubator for 24 hours. After the 24th hour, the medium was aspirated. Then, for TQ, the following working concentrations were used 5-500 µM. For Dox, 0.5–50 µM concentrations were prepared using a complete medium containing 10% FBS. The prepared concentrations were applied to wells other than the control wells in 100 µL of broth medium. At the same time, to investigate the time-dependent effect, the same concentrations were applied for 24, 48, and 72 hours. The absorbance values of the studied groups were read on a Multiskan GO microplate reader (ThermoScientific, United States of America) at wavelengths of 570 nm. To calculate the cell viability ratio and determine the IC50 value, the optical density value measured in each well divided by the control density value, the result was multiplied by 100%.

### Wound healing

The wound healing assay is used to measure two-dimensional cell migration. It is based on the creation of an artificial field called a scratch on a monolayer of confluent cells; here, the cells at the borders move towards the gap in order to fill it, and the movement is observed under a microscope. OVCAR3 cells were seeded in each well at 2×10^5^ cells. Then, a 200-μL pipette was used to scrape the cells in the middle to create a straight line, symbolizing a wound and aiding in the observation of cell migration. In this study, the trial was terminated at the 36th hour, which is when the wound was 90–100% closed in the control group, and wound healing (cell migration) was photographed in the control and application groups.

### Apoptotic body formation

In the project, nuclear morphology changes and apoptotic structures occurring after apoptosis caused by Dox and TQ were determined in the OVCAR-3 cell line with specific NucBlue Live ReadyProbes Reagent (Thermo Scientific, United States of America) dye. In this context, OVCAR-3 was planted in 24-well plates at 5×10^4^ cells. Then, Dox IC_50_, TQ IC_50_, and Dox + TQ IC_50_ were applied in the 48-hour application. Apoptotic staining was carried out directly via live cell staining in accordance with the kit’s protocol, and the cells were incubated for 30 minutes. In the last period, the plates were photographed using the Thermo EVOS FL Imaging System at 20X objective magnification.

### RNA isolation

In this study, OVCAR-3 cells were planted in 25-cm^2^ culture flasks and incubated until the logarithmic phase was reached. When the cells reached the logarithmic phase, doses of vehicle control, Dox (IC_50_ = 2.12 μM), and TQ (IC_50_ = 62.9 μM) were applied individually and in combination. RNA was isolated from the samples 48 hours after the application. A Purelink RNA mini kit (Thermo, United States of America) was used in the isolation phase, and the kit protocol was followed. Accordingly, 1% mercaptoethanol was added to the lysis solution in the kit; the medium was removed, and 1 mL of this solution was added to each 25-mL flask washed with D-PBS. These flasks were kept in a 37°C incubator for 20 minutes. At this stage, the flasks were shaken gently by hand every 5 minutes. Then, the lysed cells in the flasks were collected in 2-mL Eppendorf tubes, and 1 mL of an equal volume of 70% ultrapure ethanol (Merck, United States of America) was added to them. This mixture was vortexed and then loaded into the columns provided in the kit in 700-uL volumes. They were sequentially centrifuged at 12,000 g for 30 seconds, and the RNAs were loaded onto columns. These columns were washed first using wash solution 1 and then twice using wash solution 2. After the washing process, the columns were centrifuged for 3 minutes at 12,000 g to dry them. The columns were then placed in sterile new 1.5-mL Eppendorf tubes, 60 uL of the solution provided in the kit was pipetted into the middle of the membrane in each column, and these columns were centrifuged at 12,000 g. Pure RNAs were collected in an Eppendorf tube via 1 minute of centrifugation. RNAs were equalized to 750 ng/10 µL using ultrapure water. The concentration and purity of RNAs were determined using a Nanodrop device (Thermo). Then, a 1-µL drop of RNAse-free water was placed on the Nanodrop device’s base, and it was blindly analyzed using the analysis program on the computer (ND1000 V3.6.0). Afterwards, 1 µL of RNA sample was pipetted, and readings were carried out at 260–280 nm^6^.

### cDNA synthesis

With respect to cDNA synthesis from the isolated RNAs, a cDNA Synthesis Kit (high capacity) with an Rnase inhibitor was used as the primer and reverse transcriptase, in accordance with the manufacturer’s protocol. This is a sensitive molecular method used for the quantification of gene expression products. With this method, RNA samples can be reproduced both qualitatively and quantitatively in a shorter period of time and in larger numbers. Thus, the method allows the study to be carried out more easily with a large number of samples.

In real-time polymerase chain reaction (RT-PCR), the analysis of the products is carried out during the reaction. Therefore, processes such as electrophoresis and PCR product imaging under ultraviolet light are not necessary. In our study, an RT-PCR system capable of reading a 96-well microplate was used.

After TQ and Dox treatment, the expression levels of genes involved in proliferation and apoptosis (EGFR and FOXP3) at the RNA level were determined using the relevant cell line. Total RNA was isolated from OVCAR3 cells; then, the quantity and quality of the RNA obtained were determined. Afterwards, cDNA was performed using total RNA with a cDNA synthesis kit (high capacity) and RNase inhibitor. With this method, analyses can be performed simultaneously and quantitatively with RT-PCR (Quant Studio5). By including reference housekeeping genes in the PCR control group in each panel, we can analyze the relative change in target genes.

The amplicons obtained during PCR were evaluated according to the number of cycles that resulted in a direct logarithmic increase. First, a standard amplification curve of GAPDH and other housekeeping genes with known concentrations was created; then, the amount of cDNA was determined using quantitation software and in accordance with the transition point in the sample studied. The data obtained were recorded as Cq. The primer sequences of the analyzed epithelial–mesenchymal transition-related genes and the housekeeping gene (GAPDH) used in normalization as the reference gene are shown in Table 1.

**Table 1:** The primer sequences of the analyzed epithelial–mesenchymal transition-related genes and the housekeeping gene (GAPDH).

Primer Sequences
EGFR: F: GCCAAGGCACGAGTAACAAGC, R: GGGCAATGAGGACATAACC
FOXP3: F: GTGGCCGGATGTGAGAAG, R: GGAGCCCTTGTCGGATGATG
β-Actin: F: CCTCTGAACCCTAAGGCCAAC, R: TGCCACAGGATTCCATACCC
GAPDH: F: CGGAGTCAACGGATTTGGTCGTAT, R: GCCTTCTCCATGGTGGTGAAGAC

Source: Elaborated by the authors.

### Real-time quantitative polymerase chain reaction analysis

To determine gene expressions, EGFR and FOXP3 gene expression levels in the control and treatment groups of cells were determined via the quantitative reverse transcription polymerase chain reaction (RT-qPCR) method. RT-qPCR is used to detect and quantify RNA. Total RNA or mRNA is first transcribed into complementary DNA (cDNA). The cDNA is then used as a template for the quantitative PCR or real-time PCR reaction (qPCR). The following primers for changes in the expression of these genes were provided from 5’ end to 3’ end.

cDNAs obtained from isolated RNAs are used in gene expression studies. These cDNAs were obtained via RT-qPCR, according to the appropriate protocol, using Power Sybeer Green qPCR MasterMix (Thermo, United States of America). In this study, an Applied Biosystems Quant Studio5 RT-PCR device was used; thus, a High-Capacity cDNA Reverse Transcription Kit (Life Technologies, United States of America) was used for complementary DNA synthesis. According to the kit protocol, DNTP mix and random primers were mixed and pipetted into PCR tubes for a volume of 10 µL. Then, 750 ng/10 µL sample was equalized as described in the previous section. Total RNA was placed in the same tubes.

For the RT-qPCR reaction to occur, the following steps were carried out:

- Enzyme activation at 95°C for 10 min;
- Denaturation at 95°C for 15 s and primer binding and chain extension at 60°C for 1 min;
- A melting curve at 95°C for 15 s, 60°C for 1 min, and 95°C for 15 s.

Ct values of the peaks that emerged during the amplification process were used to identify gene expression levels, and gene expression levels were calculated via the 2-∆∆Ct method. In addition, the calibration process and multiple control methods for the endogenous control of β-actin and glyceraldehyde 3-phosphate dehydrogenase (GAPDH) mRNA expressions were provided as correction factors.

### Western blot (EGFR, FOXP3)

OVCAR-3 was planted in a 75-cm^2^ plate and held until the logarithmic phase was reached. When the control concentrations (Dox IC_50_ = 2.12 μM; TQ IC_50_ = 62.9 μM) were applied, protein isolation was performed for 48 hours after the treatment. After the medium was removed, the cells were mixed with a 500-µL RIPA lysis buffer, other homogenate buffers, and a protease inhibitor (RIPA Lysis Buffer System, sc-24948, Santa Cruz, United States of America) using a Daihan 15D tissue homogenizer (it was homogenized under cold conditions; 27,000 rpm). The homogenate obtained was centrifuged at 14,000 × g for 20 min. Protein amounts (1.2–1.6 mg/mL) were determined via the Protein A280 method using an Optizen Nano Q, Mecasys spectrophotometer. Then, proteins were loaded on NuPAGE Bis-Tris polyacrylamide gel (10%), and electrophoresis was performed.

In this study, Western Breeze brand ready-made kits provided by the Thermo Scientific company were used, and blotting and membrane transfer were carried out using the iBlot 2 (Life Technologies) system and ready-made membranes and kits following the kit’s protocols. The proteins obtained after blotting with the Anti-EGFR (Ab-1070) Antibody (ABM, CAT no.: Y021073, dilution 1:1,000), FOXP3 Antibody [C3], Cterm (GeneTex, GTX107737, dilution 1:1,000), and β-actin Antibody (Invitrogen, MA1-140) (dilution 1:5,000) were treated with primary antibodies (dilution 1:1,000). Then, the antibodies were labeled as a secondary antibody (Mouse IgG (H+L) Secondary Antibody (31,430), dilution 1:5,000) and examined using a Micro ChemiDoc (DNR Bio-Imaging Systems, USA) gel imaging system. The band intensity was calculated using GelQuant.

### Protein–Protein Interaction analysis

Protein–protein interaction (PPI) data were retrieved from the STRING database. The STRING database provides descriptions of PPIs and confidence intervals for data scores. A confidence score that was greater than or equal to 0.4 was chosen to construct the interaction network of proteins with target genes.

### Enrichment analysis

Data on the functional annotation of genes and the canonical pathways associated with the strong connections established with these proteins were obtained using the ShinyGO 0.80 program.

### Gene ontologies enrichment analysis

Three types of gene ontologies were performed on possible target genes: cellular component (CC), biological process (BP), and molecular function (MF). The SRplot bioinformatics program was used to evaluate these data.

### Statistical analysis

The difference between cell viability averages and EGFR and FOXP3 gene expressions was determined via one-way analysis of variance (ANOVA). Comparisons between paired groups were determined using the t-test or Mann–Whitney’s U test depending on the homogeneity of the data. All analyses were performed using the Statistical Package for the Social Sciences 20 (IBM, United States of America) program and according to the *p* ≤ 0.05 value.

## Results

### MTT findings

**Figure 1:**
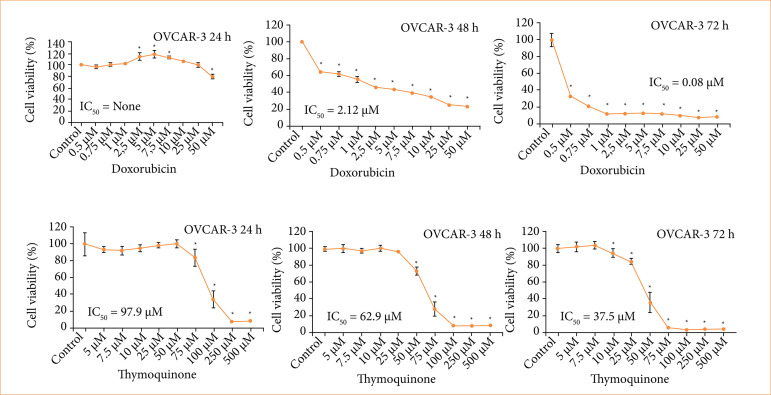
Effect of doxorubicin (Dox) applications in the concentration range of 0.5–50 µM and thymoquinone (TQ) in the concentration range of 5–500 µM on OVCAR-3 cell viability. IC50 values.

According to the scope of the study, we report the following: the results obtained via the MTT test on OVCAR-3 cells after Dox treatment; the cytotoxicity and IC_50_ values calculated using these results and obtained by applying probit analysis; and the statistical data compared to the control. As a result of these obtained data, the IC_50_ value could not be observed with respect to the 24-h Dox treatment applied on the OVCAR-3 cells. After 48 h of Dox treatment, the IC50 value was 2.12 µM, and after 72 h of Dox treatment the IC_50_ value was 0.08 µM. Significant decreases in cell proliferation were observed due to an increase in concentration. According to the statistical analysis, after the IC50 value was found, it was determined that, after 0.5-µM Dox treatment, cell viability exhibited a significant decrease compared to the control, and there was a statistically significant decrease (Fig. 1).

*Data are statistically significant compared to control, one-way analysis of variance, and Tukey’s honestly significant difference test; *p* ≤ 0.05. Source: Elaborated by the authors.

The IC_50_ value obtained as a result of TQ treatment applied on the OVCAR-3 cell line in 24 h was found to be 97.9 µM. The IC_50_ value was determined as 62.9 µM for 48 h, and the IC_50_ value obtained during the 72-h period was determined as 37.5 µM. Statistical significance between the control and TQ-treated cell groups was determined according to *p* ≤ 0.05 via one-way ANOVA and the Tukey’s honestly significant difference (HSD) test. As a result, a statistically significant decrease in cell viability was detected with respect to the 24-h TQ treatment with 75 µM concentration; 48-h TQ treatment with 50 µM concentration; and 72-h TQ treatment with 25 µM concentration (Fig. 1).

**Figure 2:**
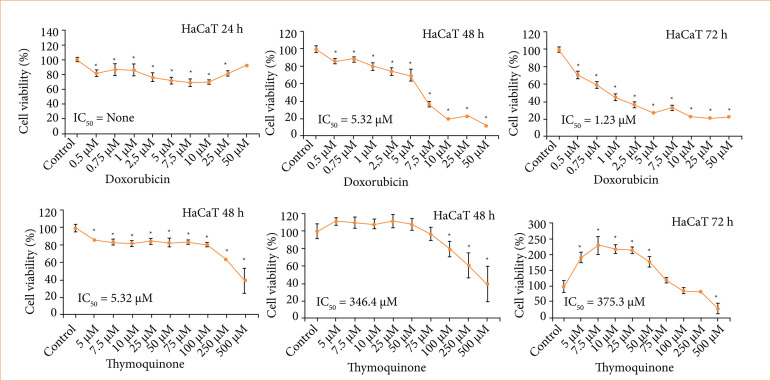
Effect of doxorubicin (Dox) application in the concentration range of 0.5–50 µM and thymoquinone (TQ) in the concentration range of 5–500 µM on HaCaT cell viability. IC50 values.

Dox and TQ treatments were applied to HaCaT cells used as healthy cells, and the results obtained were examined. As a result of the treatment with both treatment agents, % cell viability in HaCaT cells and IC_50_ values obtained via probit analysis were examined. The IC_50_ value could not be calculated in HaCaT cells with 24 h of Dox treatment. The IC_50_ value obtained with 48 h of Dox treatment was 5.32 µM, and IC50 obtained with 72 h of Dox treatment was 1.23 µM. These data showed that there was a significant decrease in cell proliferation, which was parallel to the increase in concentration. The IC_50_ value could not be calculated in HaCaT cells with 24 h TQ treatment. The IC_50_ value obtained with 48-h TQ treatment was 346.4 µM, and the IC50 value obtained with 72-h TQ treatment was 375.3 µM. These data showed that the duration–not the concentration–had a significant effect on cell viability in TQ treatment (Fig. 2).

*Data are statistically significant compared to control, one-way analysis of variance, and Tukey’s honestly significant difference test; *p* ≤ 0.05. Source: Elaborated by the authors.

**Figure 3:**
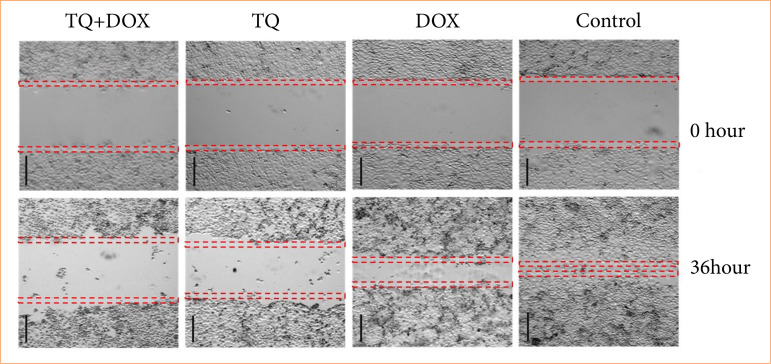
Wound healing assay in OVCAR-3 cell populations treated with vehicle control; doxorubicin (Dox) IC_50_ = 2.12 μM; thymoquinone (TQ) IC50 = 62.9 μM; TQ + Dox IC_50_ = 62.9 μM + 2.12 μM. N = 12. Bars indicate mean ± standard error.

**Figure 4:**
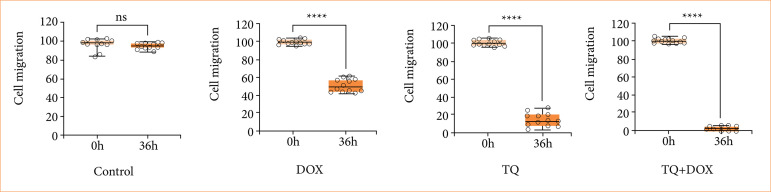
Cell migration rate and statistical significance in the wound healing model.

In OVCAR-3 cells, wound closure from the healing test was evaluated at time intervals ranging from 0 to 36 hours. Recovery in OVCAR-3 cells after the Dox-TQ application was evaluated by observing the reduction in wound size and cell migration in % compared to the control group at t = 0 (Fig. 3). When the changes in wound sizes were measured, it was observed that the wound size in the control group closed with nearly 100% cell migration after 36 h. It was observed to drop to 0 μm. In the cells treated with Dox, it was observed that the wound area, which had no cells at 0 h, was closed, with cell migration approaching 50% after 36 h (Fig. 4). In cells treated with TQ, it was observed that the wound area, which was completely open at 0 h at the beginning of the experiment, attempted to close with over 20% cell migration after 36 h. In the Dox + TQ treatment group, it was determined that cell migration for wound healing remained below 20% after 36 h.

Consistent with these results, it was determined that the application of a Dox–TQ combination resulted in decreased wound healing compared to the application of Dox and TQ alone in OVCAR-3 cells (Fig. 3).

*Data are statistically different, *p* ≤ 0.001 (magnification 10x; scale bar: 200 µm). Source: Elaborated by the authors.

***p < 0.001. Source: Elaborated by the authors.

### NucBlue staining findings

**Figure 5:**
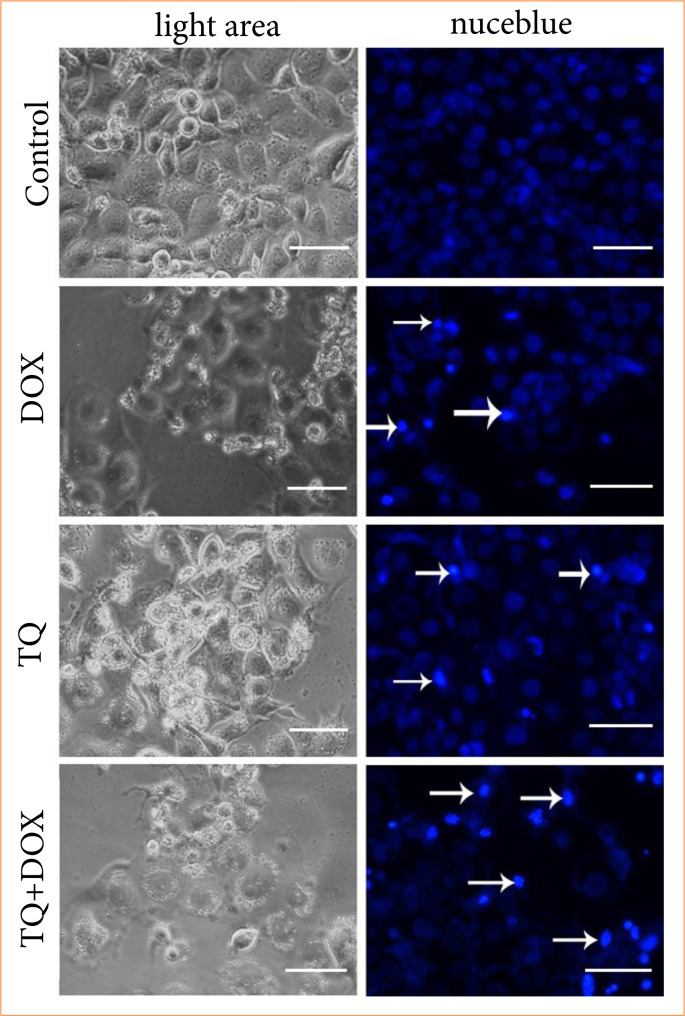
Cell morphology, nuclear structure, and apoptotic body formation in OVCAR-3 cell populations treated with IC50 concentrations of treatment agents for 48 hours (magnification 20x; scale bar: 50 µm; arrow: apoptotic cells).

NucBlue staining was performed on all samples treated with TQ and Dox in order to confirm the results of the MTT analysis and determine whether the cell deaths detected were apoptotic. It was determined that cell death increased in both TQ and Dox samples as the application concentration increased. After MTT analysis, cell viability was found to be 62.44 and 49.35%, respectively, in cultures treated with 100 µM/L TQ and 1,000 nM/L Dox. However, via NucBlue staining, it was determined that cell proliferation was suppressed above these concentrations; therefore, the number of cells decreased. Increased nuclear fragmentations indicate that apoptosis has occurred. Light microscope images also support this (Fig. 5).

Dox: doxorubicin; TQ: thymoquinone. Source: Elaborated by the authors.

Proapoptotic EGFR and FOXP3 gene expressions were normalized to the β-actin expression of the same sample, which was used as an internal control gene. EFGR, FOXP3, and β-actin gene expressions were determined at detectable levels, and amplification curves were created. The amplification curves of these genes were determined with the number of cycles on the x-axis and the Rn value on the y-axis.

**Figure 6:**
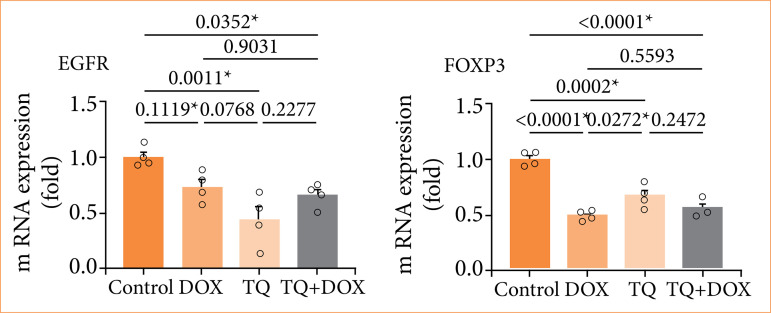
Relative folds of downregulated EGFR and FOXP3 gene expression 48 hours after single and combined drug administration (data normalized with β-actin and GAPDH mRNA levels via multiple control method; n = 4; data are shown as mean ± standard-deviation values).

As a result of the calculations, EGFR and FOXP3 were significantly downregulated in the 48-h control group. Although EGFR gene expression was determined at a detectable level in the group in which only Dox was applied for 48 h, no statistical significance could be determined between it and the control group. A statistically significant difference was detected in EGFR gene expression between the control and TQ + Dox (*p* = 0.0011) and control and TQ + Dox (*p* = 0.035) groups (Fig. 6). It was determined that the FOXP3 gene (RQ = 0.5) was expressed at the most significant level in the Dox group, with a downregulation of 50%. FOXP3 gene expression in the Dox-treated group exhibited a statistically significant difference compared to the control and TQ-treated groups. Significance was determined as p < 0.0001 between Dox and the control group, and it was *p* < 0.027 between Dox and TQ. No statistical difference was detected between the Dox and Dox + TQ groups. A significant difference was detected in FOXP3 gene expression in the TQ-treated group only between the control and Dox groups. No significance could be detected when compared to other groups. A statistically significant difference was detected in FOXP3 gene expression between the control and the Dox + TQ group (*p* = 0.0001) (Fig. 6).

*Means are statistically different. Horizontal lines indicate statistical comparison and significance between groups; TQ: thymoquinone; Dox: doxorubicin. Source: Elaborated by the authors.

### Western blot findings

**Figure 7:**
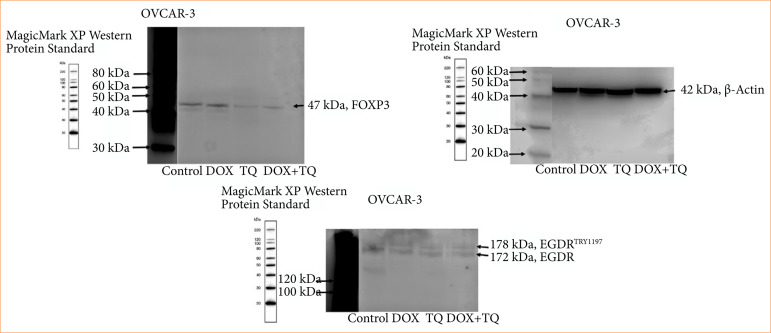
p_EGFR, EGFR, and FOXP3 protein levels in OVCAR-3 cell populations treated for 48 hours with IC_50_ concentrations of therapeutic agents.

TQ: thymoquinone; Dox: doxorubicin. Source: Elaborated by the authors.

TQ and Dox-induced apoptosis and inhibitory cell proliferation were analyzed at the protein level. As described in Fig. 7, the p-EGFR and protein expressions of EGFR and FOXP3 were found to be significantly inhibited by 48 h IC50 TQ and Dox treatment.

### Protein–protein interaction analysis

**Figure 8:**
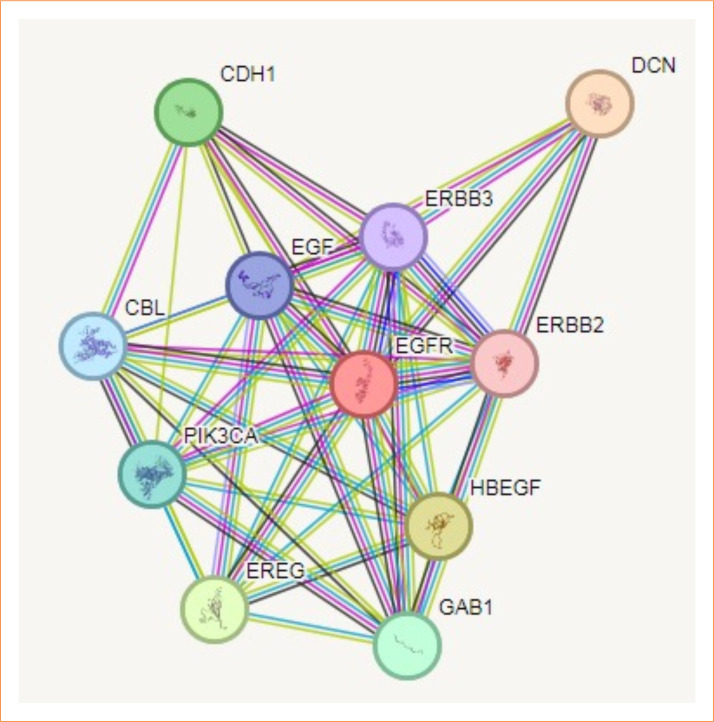
Protein–protein interaction and interaction between various genes of ovarian cancer.

Source: Elaborated by the authors.

Predictions from STRING analysis were used to depict protein interactions. The visualization exhibits 11 nodes and 46 edges (Fig. 8). Based on the nodal degree, the following genes were identified as the top 10 central genes: *DCN, CDH1, ERBB2, ERBB3, EGF, CBL, PIK3CA, EREG, GAB1*, and *HBEGF*. These targets are hypothesized to be the primary targets in TQ ovarian cancer.

### KEGG enrichment analysis

**Figure 9:**
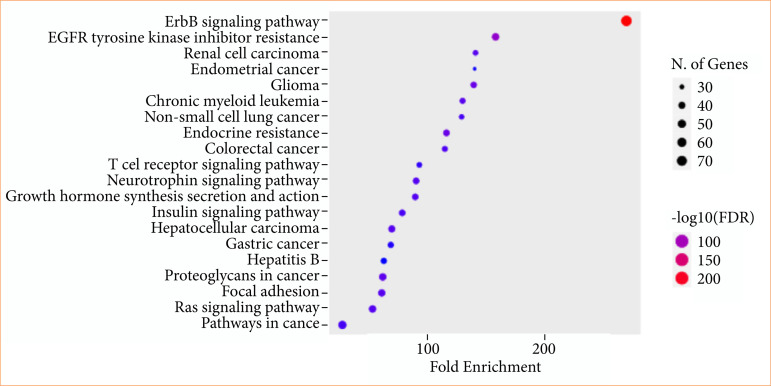
Enrichment analysis of the 72 common compound targets. In total, 50 of these targets are directly related to cancer signaling pathways, while the number of targets associated with colorectal and renal cancers is less than 30.

Source: Elaborated by the authors.

KEGG enrichment analyses of target genes were performed with the Shiny 0.80 program. The findings showed that 72 genes were involved, and 50 pathways were cancer-related, exhibiting a significant correlation with target genes (*p* < 0.05). The ErbB signaling pathway, EGFR tyrosine kinase inhibitor resistance, renal cell carcinoma, endometrial cancer, etc., are shown in Fig. 9.

### Gene ontologies functional enrichment analysis

**Figure 10:**
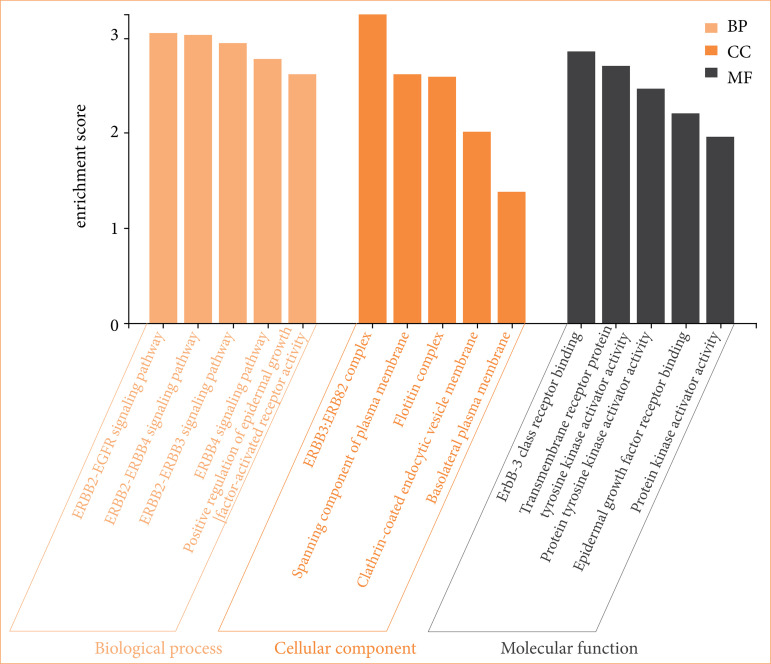
Gene ontologies (biological process, molecular function, and cellular component) analysis.

Source: Elaborated by the authors.

The analysis findings only showed important functions (Fig. 10). Target genes were found to be involved in various cellular components in the BP category, such as the ERBB2-EGFR signaling pathway. In terms of CC, target genes have been implicated in Neuroglin receptor activity, EGFR activity, etc. It was observed that the MF category exhibited different roles, such as those that involve the ERBB3-ERBB2 and fotilin complexes (Fig. 10).

## Discussion

The increase in the mortality rate of cancer is increasing every year and causes the death of hundreds of thousands of people. Unfortunately, standard treatments such as chemotherapy drugs, radiotherapy or immunotherapy, which are becoming more and more important every day, are only successful in a portion of patients due to the high heterogeneity of the disease. In gene therapy or treatments developed for molecular targets, a single gene or a product of that gene or the signaling pathway responsible for the expression of that gene is targeted to eliminate a specific group of cells in the tumor.

The mechanisms that result in the formation and development of many types of cancer are completely different, and the underlying causes are often not fully understood. However, it is thought that changes in the genetic and epigenetic pathways of different cancer types result in the emergence of cancer^14^.

Based on this information and the development of different cancers, surgery, chemotherapy drugs, radiotherapy, immunological modulation, the targeting of non-coding RNAs, and various hormonal therapies are used for cancer treatment^15^. Despite developments in traditional cancer treatments, clinical applications are not significantly effective due to disadvantages, such as low response to primary treatment, high probability of recurrence, organ toxicity, serious side effects, insufficient selectivity, and low quality of life^16^. Therefore, the search for modern curative procedures to combat various types of cancer with the fewest possible negative effects has become a focus of attention in recent years.

Currently, numerous studies on herbal bioactive substances are being conducted due to their low toxicity, easy availability, and affordable costs. Examining the results of these studies, TQ appears to be one of the most promising natural drugs. New studies have shown that the regulation of microRNA (miRNA) expression through TQ has been accepted as a new technique in the fight against cancer^17^. Many studies carried out on TQ have revealed that it exhibits beneficial therapeutic potential with respect to human health, especially in relation to cancer. In-vitro and in-vivo studies with different cancers have shown a broad consensus that TQ has promising anticancer properties^18^. Classical signs of apoptosis, such as DNA breaks, chromatin condensation, and phosphatidyl serine translocation, have been documented in cancer cells with TQ treatment^19^.

It is beneficial to further investigate the combination of TQ with chemotherapeutics such as Dox in order to suppress the development of multidrug-resistant tumors, including breast cancer, and increase their efficacy^20^. In a study conducted by a group of researchers with resistant breast cancer cells, it was reported that TQ and Dox increased anticancer activities in human breast cancer cells (MCF-7) and produced a significant therapeutic effect^21^. In a similar study, it was reported that TQ regulates cell proliferation and apoptosis in MCF-7 cells and increases the anticancer effect. This study showed that TQ accelerated the apoptosis of Dox-resistant breast cancer cells by upregulating transcriptional phosphatase and tensin homolog (PTEN).

Numerous studies have reported that Dox is one of the most powerful chemotherapeutics and is widely used in the effective treatment of many cancers. Breast cancer and ovarian cancer are among the cancers used. The findings showed that Dox can kill cancer cells by directly targeting DNA^22^ and induces apoptosis in cancer cells by increasing the activity of reactive oxygen species and activating the p53 signaling pathway^23^.

Black seed oil has been reported to have many medical benefits. Black cumin has many coumarins, fixed oils, saponins, volatile oils, alkaloids, nutritious proteins, minerals, and carbohydrates^24^. Dozens of studies have been conducted on TQ with respect to many different types of cancer, and it inhibited the proliferation of many tumor cells, especially ovarian carcinoma, myeloblastic leukemia, breast adenocarcinoma, colorectal carcinoma, osteosarcoma, and pancreatic carcinoma^25^. Many in-vitro studies report that TQ’s anticancer potential is achieved by suppressing the proliferation of cancer cells and directing the cells to apoptosis. In various experimental models, melatonin exhibits antioxidant properties in different tissues and organs and has been studied in cancer, neurological diseases, organ transplants, chronic diseases, and surgery. These benefits can be obtained from its direct and indirect effects on cells^26^^,^ 27. In our results, the suppression of cell viability with both an important antioxidant TQ and chemical agent Dox reflects research findings in terms of directing cells to apoptosis by activating the EGFR/FOXP3 signaling pathway. In addition, it was observed that the suppressive effect on cancer cells increased as a result of the combination with Dox.

In recent studies, it was reported that TQ can increase survival in colorectal cancer treatment. With respect to the effect of TQ on colorectal cancer cells, it was shown that it plays a role in the emergence of antitumor effects as a result of the disruption of MAPK 7 and MAPK 1 TQ treatment-suppressed cancer cells with a direct antitumor effect, and it also rendered them sensitive to other treatments^28^. Another study^29^ reported that human breast carcinoma cells were suppressed via TQ treatment by making them radiosensitive. In addition, in a study conducted on lung cancer cells^30^, it was stated that TQ treatment eliminated resistance and rendered them sensitive to the chemotherapeutic agent cisplatin. On CAOV-3 ovarian cancer cells, TQ decreased the permeability of plasma and mitochondrial membranes; inhibited Bcl-2 and Bax, which are regulators of the apoptosis pathway; decreased the nuclear area; induced apoptosis in ovarian cancer; and increased oxidative stress^31^. In another study, a higher apoptosis rate was achieved with the combination of TQ with cisplatin, and it was shown that this led to better results when the Bax/Bcl-2 ratio was increased^32^. Johnson-Ajinwo et al. emphasized the importance of examining the cancer of TQ^33^. It was shown that, with the combination of TQ and cisplatin, DNA breaks increased; therefore, apoptosis increased, and proliferation decreased^34^. It was reported that TQ has a high probability of inducing apoptosis in osteosarcoma and can also reduce the number of cells in the S phase. The findings obtained in all these studies conducted on different cancer cells have similar characteristics. In this study, apoptosis was induced via EGFR and FOXP3, inhibiting the proliferation of OVCAR3 and increasing the number of cells undergoing apoptosis.

Cancer is now a deadly disease that kills hundreds of thousands of people every year. By targeting a single gene, gene product, or signaling pathway, only a certain group of cells in the tumor can be eliminated, while other genetically distinct variations can easily escape treatment. This situation could result in drug resistance in cancers^28^^,^ 35 ^,^ 36. All these negative consequences are caused by genomic instability and the aberrant activation of genes that detect and repair DNA damage. Platinum-based chemotherapies are applied as sensitizers of genotoxic therapy to improve the failure of cancer treatments by inhibiting DNA damage repair and also causing cell death^37^^,^ 38.

To observe the effectiveness of drug redirection in ovarian cancer, we applied a combination of Dox and TQ, each of which acts as an adjuvant therapy in cancer treatment. Considering this information, whether the Dox-TQ combination could be a new applicable cancer treatment drug was investigated. The cytotoxic effects of Dox and TQ alone or in combination with OVCAR3 and HaCaT cells were evaluated in the MTT test for 24, 48, and 72 hours. According to the effects of TQ and Dox application on cell growth rate, it was observed that the cell growth rate of cancer cells decreased depending on the time and concentration at 24, 48, and 72 hours.

This is also the first study to determine the effect of TQ-Dox application on the growth rate of OVCAR3 and HaCaT cells. According to the results, the greatest cytotoxic effect of TQ alone on the cell viability of OVCAR3 cells was observed after 72 hours, and the IC_50_ value was 37.5 μM. TQ cytotoxicity was determined in PC-3, LNCaP, and DU145 cell lines by Zubair et al.^39^ and Jafri et al.^40^. In these studies, IC_50_ values = 55.3 μM and 50 μM TQ were determined in pancreatic cancer cell lines^39^^,^ 40. After 72 hours, the highest cytotoxic effect of TQ applied alone on HaCaT cell viability was observed with an IC_50_ value of 375.3 μM. In this study, OVCAR3 cancer cells were found to be more sensitive to TQ alone and in combination with others compared to HaCaT cells.

The greatest cytotoxic effect of Dox alone and in combination with others on the cell viability of OVCAR3 cells was observed after 72 hours, and the IC_50_ value was determined as 0.08 μM when Dox is applied alone. On the contrary, the effect on HaCaT cells with respect to Dox treatment was found to be 1.23 μM. In a similar study, Alekseev et al.^41^ showed that spiro chemosensitizes cancer cells relative to platinum compounds. The authors performed two experiments: an experiment combining spironolactone with oxaliplatin and cisplatin compounds in ovarian carcinoma cells A2780 and HCT-116; and an experiment with only oxaliplatin and cisplatin compounds. They reported that the IC50 value was more sensitive in the presence of spironolactone and platinum. Finally, they stated that spironolactone chemosensitizes different cancer cells relative to platinum-based anticancer agents by inducing the degradation of XPB in these cells^41^. It could be concluded that, in this study, the compound Dox increased cytotoxicity in OVCAR3 cells by producing a synergistic effect with TQ in a time- and dose-dependent manner.

A wound healing assay was performed to examine the effects of TQ and Dox alone and in combination on the migration of OVCAR3 cells. The IC_50_ values obtained via the MTT assay were used to observe the effect of the agents on cell migration. According to the results of the cell migration assay, it was determined that Dox and TQ alone and in combination inhibited the migration of OVCAR3 cells after 36 h. Finally, the greatest inhibition of OVCAR3 cell migration was determined in the combination treatment. Similar studies have shown that it inhibits the migration of different cancer cells, but we could not find a study carried out on the effect of its combination with Dox on cancer cells^42^^–^ 44.

Upregulated EGFR expression is associated with poor prognosis in cancers and has been highly expressed in ovarian cancer, triggering poor prognosis. A study conducted on cancer cells has shown that bufalin reduces EGFR protein and expression levels, and the downregulation of AKT and ERK and phosphorylation levels is carried out via bufalin stimulation^45^. EGF is fixed to the extracellular domain of EGFR and induces EGFR dimerization^46^. EGFR amplification or its high expression in ovarian cancer plays an important role in the prognosis of the disease. In one study, it was proven that bufalin can bind to EGFR via the molecular docking method and thus suppress the proliferation of ovarian cancer cells. Moreover, protein levels determined via Western blot confirmed that the EGFR/AKT/ERK pathway stops the proliferation of cancer cells. However, in studies investigating the effect of bufalin on lung cancer cells immediately afterwards, it was shown that it was effective by inhibiting the phosphorylation of the EGFR protein without affecting EGFR protein levels^47^. As a result, targeting the EGFR signaling pathway in different cancer cells and discovering suppressive agents are important. We were able to suppress the proliferation of ovarian cancer cells with TQ by carrying out molecular regulation that targeted EGFR.

Despite the promising findings from this study for cancer treatment, several limitations should be acknowledged. The first limitation is that the study was conducted in a single cell line. This may disrupt the later stages of ovarian cancer and may not fully represent the complexity in vivo. In addition, the study focused primarily on the EGFR/FOXP3 signaling pathway, leaving other potential anticancer pathways affected by TQ unexplored. In-vivo studies including more comprehensive molecular parameters are needed to elucidate the therapeutic potential of TQ with Dox in ovarian cancer.

## Conclusion

RT-qPCR and protein expression results showed that TQ and Dox can provide active treatment in ovarian cancer via the EGFR and FOXP3 signaling pathways. It was observed that TQ can inhibit tumor cell growth. Its mechanisms and conditions need to be determined via in-vivo studies. It was also observed that the combination of TQ and Dox may be an alternative and a new treatment option in cancer treatment.

## Data Availability

All data supporting the findings of this study are available in public databases such as PubChem and STRING and in the paper. The original contributions presented in the study are included in the article, further inquiries can be directed to the corresponding author.
